# Contribution of nutrient fluxes to the evolution of the net energy systems, example of the INRA feeding system for beef cattle

**DOI:** 10.1093/tas/txz074

**Published:** 2019-06-25

**Authors:** Isabelle Ortigues-Marty, Jacques Agabriel, Jean Vernet, Bernard Sepchat, Marwa Al-Jammas, Pierre Nozière

**Affiliations:** UMR1213 Herbivores, INRA, Vetagro Sup, Saint-Genès-Champanelle, France

**Keywords:** carcass composition, diet, fattening cattle, feed energy value, feeding system, partition of nutrients

## Abstract

Energy feeding systems define energy as a whole, but progress made to define metabolizable energy (ME) as the sum of the metabolizable nutrients produced by digestion and available for tissue metabolism in a wide range of nutritional situations opens the way to quantitatively model and predict nutrient fluxes between and within tissues and organs and improve predictions of energy use. This review addresses the contribution of nutrient flux concepts and data to the evolution of the Institut de la Recherche Agronomique (INRA) energy feeding system for growing and fattening cattle and evaluates the outcomes on the net energy (NE) requirements. It summarizes recent progress made to quantitatively predict nutrient fluxes both at digestive and visceral levels. It reviews how nutrient flux concepts and results were introduced in the recently updated INRA feeding system, resulting in improvements in the accuracy of the revised digestible energy (DE) and ME value of diets. The use of an independent database showed that for diets fed to fattening cattle, DE and ME concentrations were downgraded for low-energy-dense diets and upgraded for high-energy-dense diets. We are also showing that compared with its previous version, the updated INRA system improves the quantitative relationship between ME supply and flows of metabolizable nutrients. Evidence is provided on how measured nutrient fluxes at portal level were used to evaluate the predicted flows of metabolizable nutrients. This review then revisits the NE values of diets for fattening cattle as defined by the INRA feeding system and not updated yet. Using an independent database and at similar ME intake, carcass composition was shown to be linearly related to the energy density of diets for fiber-rich diets but not for concentrate-rich diets, suggesting that the efficiency of energy utilization of ME into NE is not linearly related to differences in the composition of the gain. Accounting for the balance of metabolizable nutrients or their proxies in models used to predict carcass composition from ME intake can improve predictions. Overall partitioning aggregated energy fluxes into their subcomponent nutrient fluxes in a more physiological approach offers promising perspectives for the evolution of NE feeding systems.

## INTRODUCTION

Feeding practices of livestock are facing new nutritional challenges besides optimizing performances, such as reduce losses to the environment, sustain animal health, and improve production and quality of products. Feeding systems, that is, models that predict nutritional supply to and requirements of the animals in similar units (e.g., fill unit, metabolizable protein [MP] and amino acids, digestible, metabolizable, or net energy [DE, ME, or NE], and absorbable minerals), are expected to address these current and new production challenges. Animal scientists share the conviction that this is possible if the newly acquired knowledge, and hence probably greater complexity, is introduced in the existing models. This involves refining the feeding systems, as done previously on a regular basis when new knowledge becomes available to overcome the limits of the existing systems.

In NE feeding systems, knowledge is aggregated at the whole animal level and formalizes the exchange of nutrients between the animal and its environment. Nutrients are considered in groups and defined using aggregated units of energy, such as gross energy (GE) which is itself partitioned into fecal, urinary, gas, and heat losses, thereby defining DE, ME, and NE. Partition of energy follows the first law of thermodynamics of energy conservation and sums always add up (Blaxter, 1962). The first energy researchers used it to develop concepts of energy use (Brody, 1945; Kleiber, 1961) that formed the basis of the development of energy feeding systems. Also energy exchanges are considered over fixed periods of time and the day is the usual time unit. Conversion coefficients between the different levels of energy do not, however, cover all possible conditions in practice (animal types and variety of diets), and new challenges are not necessarily addressed. For example, for beef cattle, performances and quality of production (e.g., carcass composition) may not always be accurately evaluated.

The current approaches to update the NE feeding systems, however, differ. Debate exists on the level of complexity to introduce and on the optimum balance between complexity and pragmatism required for a system to be applied in practice for different productions (e.g., beef and dairy). We consider that the concept of nutrient flux is helpful to define the next level of complexity to add and select the lower level(s) of knowledge to aggregate. The concept is not new, it refers to energy partition. Strictly, nutrient flux refers to the rate of flow of metabolites (e.g., the product of nutrient concentration and the rate of flow of the biological fluid under focus) in and out of a body compartment or the movement of a metabolite across body compartments. The concept of nutrient flux, hence, represents the nutrient transactions either between the animal and its environment or between compartments at tissue or cellular levels within the animal. It reflects the outcomes of the complex and interdependent metabolic relationships between nutrients within and between compartments.

To apply nutrient flux concepts to the NE feeding systems implies to partition the aggregated energy fluxes into their subcomponent fluxes in a top-to-bottom approach and make sure that the sums add up. Lindsay (1993) strongly pleaded for it. It means moving from joules to moles of molecules or groups of molecules. Evaluating ME as the sum of the metabolizable nutrients produced by digestive processes and available for tissue metabolism has been an early goal (see, e.g., Journet and Huntington, 1995, for review), but the process has been hampered by the limited number of data available. Subdividing current energy fluxes at whole animal level into their component fluxes in a quantitative manner requires access to sufficient reliable data that cover a wide range of animal and nutritional conditions. In ruminants, data are now available from the nutritional studies published worldwide over the last five decades that measured digestive flows and metabolizable nutrients. The data reported at group-average level can be assembled into databases and analyzed with standardized methods. When several complementary databases are used, response equations derived from one database can be tested against data or response equations from other databases. This step forward has already been made for MPs that have been defined as metabolizable amino acids, enabling imbalances among amino acids to be addressed.

The perspective to define ME as the sum of the metabolizable nutrients produced by digestive processes and available for tissue metabolism in a wide range of nutritional situations opens the way to quantitatively model and predict nutrient fluxes between and within tissues and organs. Measurements of blood/plasma nutrient fluxes across tissues or organs have also been widely investigated over the last five decades (the principles of which can be found in Bergman et al., 1970 and have been reviewed in Huntington and Reynolds, 1987; Seal and Reynolds, 1993; and Reynolds, 2002) and produced a bulk of data assembled in the Flora database (Vernet and Ortigues-Marty, 2006). Data are now sufficient to evaluate nutrient fluxes in and out of a tissue or organ, reducing the black box from the whole animal down to the tissue and organ level.

This review addresses the contribution of nutrient flux concepts and data to the evolution of feeding systems, using the Institut de la Recherche Agronomique (INRA) energy feeding system for growing and fattening cattle as an example. It summarizes recent progress made to quantitatively evaluate nutrient fluxes both at digestive and metabolic levels. It reviews how nutrient flux concepts and results were empirically introduced in the recently updated INRA NE feeding system to revise the ME value. It evaluates the outcomes of the update and revisits the NE values of diets. The review does not present an exhaustive analysis of the literature. It highlights the ever-living relevance of the concept and of its top-down development, starting by the most aggregated level of energy supply and decomposing it into its fractions. The review is limited to energy for growing and finishing cattle.

## WHY CONSIDER NUTRIENT FLUXES? EXAMPLES OF LIMITS TO CURRENT AGGREGATED ENERGY VALUES OF RATIONS

**Figure 1.**
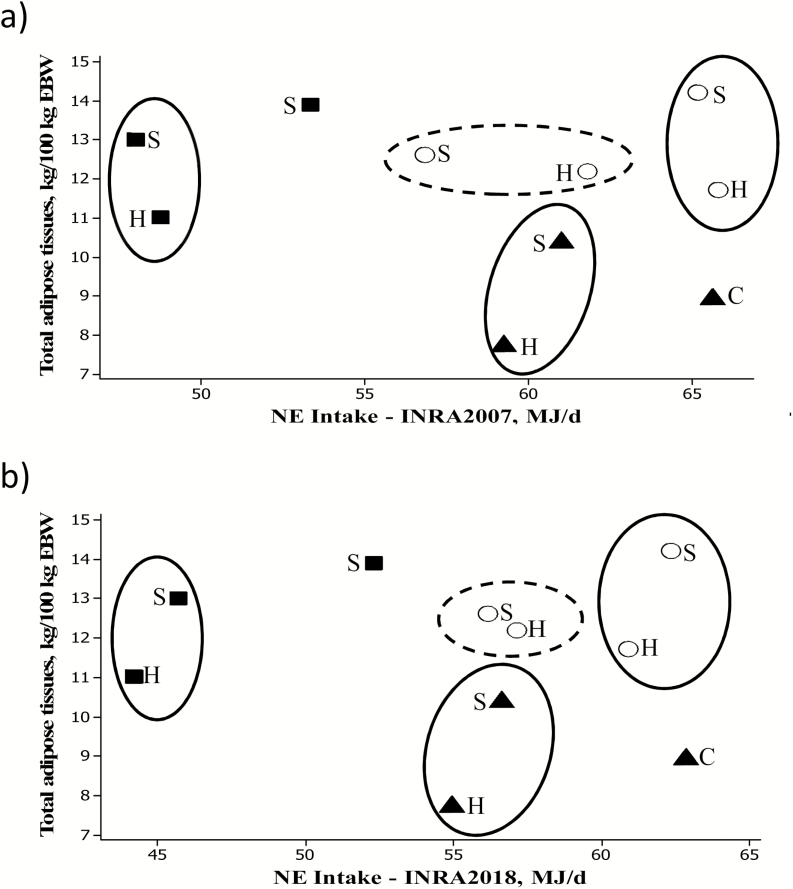
Relationship between total adipose tissues in the empty body weight (EBW) of fattening cattle (three different Continental beef breeds) and NE intake evaluated from (a) INRA (2007) and (b) INRA (2018). Each point is a treatment mean. Data were obtained from Geay et al. (1997, ■), Micol et al. (2007, ▲), and Sepchat et al. (2013, ○); animals were fed corn or grass silage (S), hay or haylage (H), or concentrate (C) rich diets.

The use of nutrient fluxes emerged as an alternative to address some of the current limits of the aggregated energy value of rations. In a context where society is putting pressure on producers to feed ruminants with nonhuman edible feeds and an increasing proportion of grass or forages, researchers are questioned on the impacts of different diet composition at similar NE intakes. A set of three feeding trials conducted at INRA Theix using young fattening bulls of different breeds and designed to feed animals at isoenergetic NE levels was recently revisited (Figure 1a). In each trial, animals were individually fed the same amount of NE and MP per day but from different diets based on hay, haylage, grass silage, or corn silage. Animals were slaughtered either at the same age (Geay et al., 1997; Sepchat et al., 2013) or at the same weight (Micol et al., 2007). Intriguingly, neither the growth performances nor the carcass composition were related to NE intake. Within study, isoenergetic NE supply resulted in different total adipose tissue masses per unit of empty body weight, while different NE intake resulted in similar total adipose tissue masses. Overall, animals fed the corn or grass silage diets had significantly higher growth rates and a higher proportion of adipose tissues in the carcass than those fed the hay or haylage diets. Similar observations had also been made in farm conditions (J. Agabriel, personal communication). Authors speculated that the NE values of the silage diets were underestimated because intake of silages had to be restricted to meet iso-NE intakes (Geay et al., 1997) or because of digestive interactions (Micol et al., 2007) or that the metabolic use of rations differed (Sepchat et al., 2013). Such examples questioned the accuracy of 1) the evaluation of ME intake and 2) the prediction of energy deposited during growth (NE) or lost as extra heat. These two questions are addressed below in light of current progress made using nutrient flux concepts. The first section describes how digestive fluxes were applied in INRA (2018) to improve the accuracy of the evaluation of dietary ME concentrations. The second section describes recent progress to partition ME in nutrient fluxes. The final section presents the ongoing work made to revisit the conversion of ME into NE using nutrient flux concepts and results at tissue levels and offers perspectives to integrate nutrient fluxes in future updates of feeding systems.

## REVISITING THE DIETARY ME VALUES

### Overview of Dietary ME Values in Different Feeding Systems

The aggregated ME unit of feeds and diets, as well as the initial GE and DE values, are predicted differently across feed evaluation systems. First, the GE content of feeds is derived from their chemical composition (INRA, 2007; Volden, 2011, VEM [feed unit for milk production according to the Dutch system], i.e., Van Es, 1978), whereas NRC (2001) directly considers the concept of digestible nutrients. The subsequent transformation of GE into DE and ME depends on diet digestibility and energy losses as methane (CH4) and urine. In the first versions of the INRA feed evaluation system (INRA, 1978, 2007) DE is a simple but key variable. It is based on extensive in vivo measurements of organic matter (OM) digestibility in sheep. By contrast, in NRC (2001), DE is calculated as the sum of GE from the apparently digested organic fractions (neutral detergent fibre [NDF], non-fiber carbohydrates, crude protein [CP], fat), each of these digestible fractions being estimated from chemical composition, whereas it is ignored in Volden (2011) and VEM (Van Es, 1978). The ME content of feeds is then directly derived from DE (NASEM, 2016) and the chemical composition of feeds (NRC, 2001; INRA, 2007) or from the OM (or organic compounds) digestibility and the chemical composition of forages (VEM Van Es, 1978). Alternative calculations exist by summation of all digestible fractions estimated either from chemical composition (VEM, i.e., Van Es, 1978, for concentrates) or from a mechanistic digestive model (Volden, 2011). In INRA (2007), energy losses as urine and CH4 are subtracted from DE to determine ME. These energy losses are determined from a ME to DE ratio and not separately. Furthermore, the intake level used to measure or calculate DE or ME varies widely among systems. It can be variable between feedstuffs (i.e., corresponding to ad libitum intake of forage in INRA, 2007) or fixed and common to all feeds (2.38 times maintenance in VEM (Van Es, 1978), the actual animal production level in Volden (2011), or three times maintenance in NRC (2001)). This implies that for a given feedstuff, ME value varies among systems. The accuracy of the DE and ME concentrations of feeds and diets depends on the evaluation of the diet digestibility and energy losses as CH4 and urine. In INRA (2007), estimation of energy losses as urine and CH4 did not account for the feeding level; they were kept aggregated even though they do not have the same physiological origin nor the same environmental impact.

### Introduction of Digestive Fluxes in the INRA (2018) Update of Dietary ME Values

Because the INRA (1978, 2007) feeding system proved its robustness and relevance in practice, the approach was to update and improve it rather than to develop a new one. Hence the new results that have been introduced are always linked to long established concepts and models and are based on robust average within-experiment response laws of digestive events to feeding practices.

**Figure 2.**
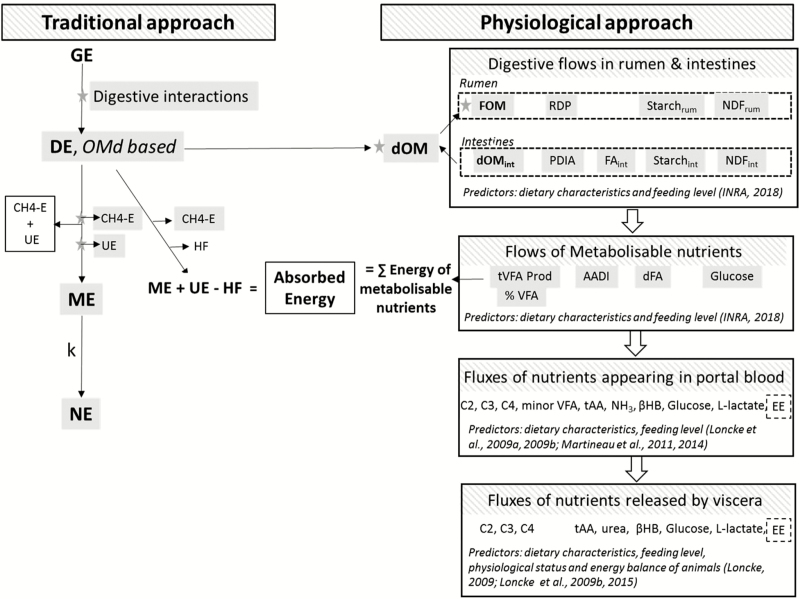
Combining energy fluxes at whole animal level with nutrient fluxes at digestive and visceral levels defined according to a physiological approach. Gray shadings highlight INRA (2018) novelties. Abbreviations: (t)AA(DI), (total) amino acids (digestible in the intestine); βHB, β-hydroxybutyrate; C2, acetate; C3, propionate; C4, butyrate; dFA, digestible fatty acids; k, efficiency of ME use into NE; HF, heat of fermentations; dOM(int), digestible OM (in the intestine); EE, energy expenditure; FAint, fatty acids digestible in the intestine; NDF_rum_, NDF_int_, NDF digestible in the rumen or in the intestine; OMd, organic matter digestibility; PDIA, protein digestible in the intestine of dietary origin; RDP, rumen degradable protein; starch_rum_, starch_int_, starch digestible in the rumen or in the intestine; UE, urinary energy; (t)VFA (Prod), (total) volatile fatty acids (production); %VFA, molar proportion of volatile fatty acids

The 2018 revision of the INRA feed evaluation system first addressed diet digestibility (Figure 2). The latter is known to be nonadditive, but digestive interactions are not systematically nor homogenously accounted for in all systems. In INRA (2007), DE values of rations were not corrected for digestive interactions, instead a correction was applied on the NE value of rations for dairy cows or were empirically included in the NE value of corn silage diets and in the NE requirements of beef cattle or dairy goats. The INRA (2018) approach was to evaluate digestive interactions at DE (and hence OM digestibility) level for a closer fit to biology. The main factors influencing diet digestibility were identified, quantified, and ranked. By principle, the range of factors influencing diet digestibility was restricted to easily measurable diet characteristics, considered as proxies of finely tuned digestive processes. Indeed, although the evolution of feed evaluation systems toward more precise prediction of nutrient supply theoretically requires more and more sophisticated indicators on feedstuffs, the cost of any systematic characterization requires pragmatism. On those bases, the main factors responsible for digestive interactions and influencing DE values of rations were found to be 1) the feeding level (in kg dry matter intake [DMI]/100 kg body weight [BW]), which was linearly related to the passage rate of digesta (separated as forage particle vs. concentrate particle vs. liquid phase and expressed in %/h) through the rumen, 2) the proportion of concentrate in the diet, which curvilinearly affects the rumen pH and the activity of cellulolytic microorganisms, and 3) the rumen protein balance which evaluates the availability of nitrogen for microbial activity. It was checked that the impacts of these three characteristics were fully additive. A major consequence of those changes is that a feed does not have a unique DE value anymore, its value varies with diet composition and intake level. This concept has also been retained in NRC (2001) or Volden (2011), where standard feed values are given for several intake levels. Of particular relevance for beef cattle, and whereas in INRA (2007), digestive interactions were mainly taken into account through an empirical correction of the tabulated NE value of corn silage, INRA (2018) explicitly integrates in a factorial way the effects of feeding level and digestive interactions between feedstuffs within a ration. The effects of urea or rumen degradable N, supplementation on corn silage digestibility, and energy values of corn are also included.

INRA (2018) also proposes a simple approach to dissociate urinary from CH4 energy losses (Figure 2), using indicators already used in DE or MP calculations. Urinary losses can be predicted from the dietary CP content and from the feeding level and the proportion of concentrate in the diet, both factors affecting the balance between CP degradability and available N utilization for microbial growth, thus the rumen protein balance. Methane losses per kg digestible organic matter, only depend on the feeding level and the proportion of concentrate in the diet according to quadratic relationships, reflecting that fermentation pathways used with high intake and high concentrate diets produce less adenosine triphosphate and CH4 per C fermented toward volatile fatty acids (VFA) (Sauvant et al., 2011). Cornell Net Carbohydrate and Protein System (CNCPS) proposes an estimation of CH4 derived from ME intake, although not included in the calculation of ME, and dietary chemical composition (Van Amburgh et al., 2015). Others adopt a more mechanistic approach where CH4 is computed from the other end products of fermentation in the rumen (Tedeschi and Fox, 2018). NASEM (2016) provides both empirical and mechanistic equations for CH4 emissions.

### Consequences of INRA (2018) Update on the ME Values of Rations for Fattening Young Bulls

To evaluate the outcomes of INRA (2018) on the nutritional values of diets for beef cattle, we used a database, Alicar, built from studies published in international peer-reviewed journals (Vernet et al., 2016). Alicar gathers data from studies researching the impact of dietary conditions on growth and carcass composition of young fattening cattle that had been fed a similar diet postweaning. Trials that fed animals with different diet compositions during the growth and the fattening periods were excluded. Eighty-three publications were used for a total of 437 experimental treatments, and data refer to group averages per treatment. Animals were characterized according to a typology on breed origin (Continental, British, and Dairy), production purpose (meat, milk, and dual purpose), maturing rate (from early to late), frame size, and sex as described by Al-Jammas et al. (2016). Diet ingredients were described according to INRA feed tables (2007 and 2018) as in Loncke et al. (2009b). Alicar included a wide range of experimental conditions, DMI varying between 5.2 and 13.3 kg/d, average daily gain (ADG) from 212 to 2130 g/d, and the proportion of lipids in the carcass from 21% to 38%.

**Figure 3.**
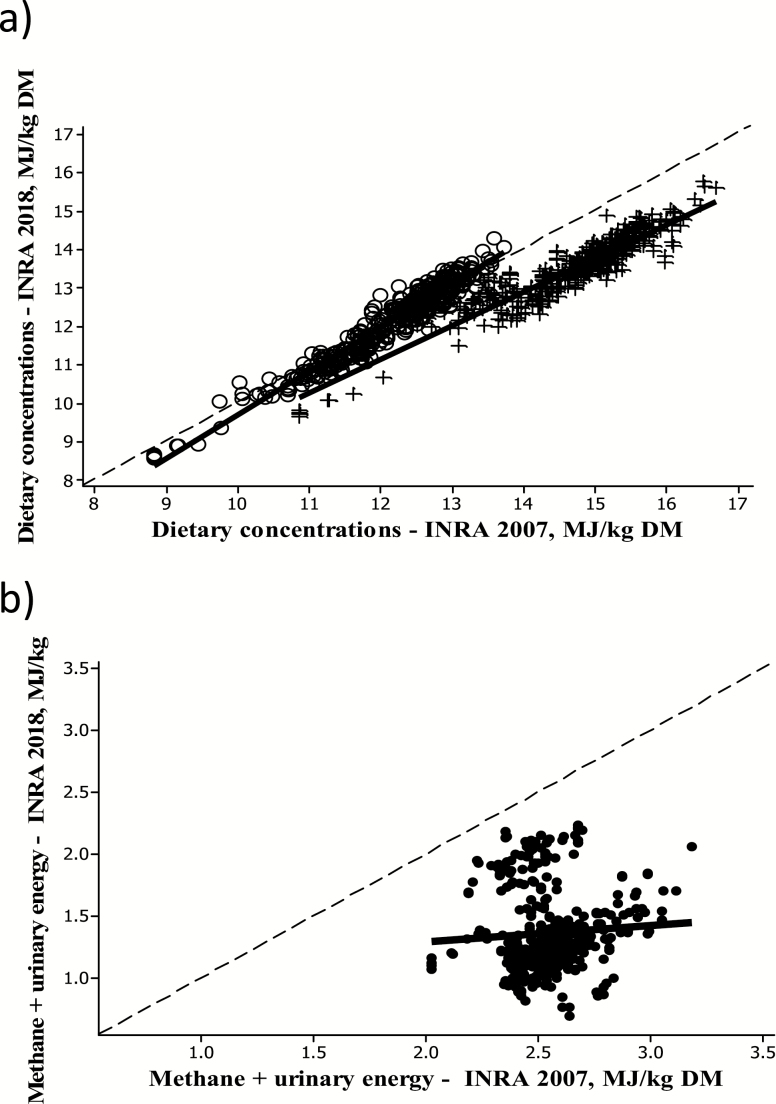
Relationships between (a) dietary concentrations in DE (+) and ME (o) and (b) methane and urinary energy losses evaluated from INRA (2007, 2018) using the Alicar database, all in MJ/kg DM. ME_2018_ = −1.60*** (0.17) + 1.13*** (0.01) ME_2007_, *r*^2^_adj_ = 93.5%, residual mean square error (RMSE) = 0.25. DE_2018_ = 0.67** (0.25) + 0.87*** (0.02) DE_2007_, *r*^2^_adj_ = 86.17%, RMSE = 0.33. (CH4+UE)_2018_ = 1019.22*** (235.56) + 0.14^NS^ (1.46) (CH4+UE)_2007_, *r*^2^_adj_ = 0.26%, RMSE = 330.30.

On average, the dietary DE concentrations tended to decrease for all diets by an average of 8% as a result of digestive interactions between forage and concentrates and the level of DMI (Table 1). In Alicar database, intake ranged from 1.3% to 3% BW in fattening cattle, whereas in INRA (2018), OM digestibility and hence DE concentrations of forages are measured at reference intake levels around 1.6–2.1 (grass silages), 1.4 (corn silage), or 1.6–2.5 (hay) and that of concentrate at 2% BW. The decrease was thus greater for high-energy-dense diets fed at high intake levels (Figure 3a). The drop in dietary DE concentrations was, however, compensated by energy losses as CH4 and urine being significantly reduced by an average of 46%. The drop in CH4 and urinary losses was also greater for high-energy-dense diets (Figure 3b).

**Table 1.** Animal and diet characteristics according to INRA (2007) and INRA (2018) in the Alicar database (83 publications, 437 treatments)

	INRA (2007)				INRA (2018)			
	Mean	SD	Min	Max	Mean	SD	Min	Max
Animal								
BW, kg	436	66.99	233	583				
DMI, kg	9.08	1.66	5.21	13.33				
DMI/BW, %	2.09	0.32	1.35	3.03				
Diet composition								
Concentrate DM/total DM	0.79	0.24	0.00	1.00				
OM, g/kg DM	941	15.1	884	980				
CP, g/kg DM	141	27.8	73	286				
Diet digestibility								
OMd, %	83	5.0	63	89	76	4.2	56	84
Ed, %	80	4.9	61	87	73	4.3	54	82
Nutritional value of diets								
DE, MJ/kg DM	14.7	0.94	10.8	16.7	13.5	0.88	9.63	15.76
ME, MJ/kg DM	12.2	0.84	8.8	13.7	12.1	0.99	8.6	14.3
*q* (ME/GE)	0.66	0.045	0.50	0.73	0.66	0.058	0.48	0.74
MP, g PDI/kg DM	94	15.7	46	149	91	10.7	61	128
Evaluation criteria								
FOM, g/kg DM	517	64	404	757	528	51.3	393	697
RPB, g/kg DM					−0.08	22.14	−49.41	117.37
Predicted energy losses to environment								
CH4-E, kJ/kg DM					805	243	363	1,436
UE, kJ/kg DM					558	126	316	1,179
Total losses, kJ/kgDM	2,534	170.6	2,023	3,176	1,363	330.7	688.9	2,228

CH4-E: energy loss as methane; d, digestibility; OMd, organic matter digestibility; RPB, rumen protein balance; UE, urinary energy loss.

As a consequence, the INRA (2018) revision did not significantly modify the dietary ME concentrations of Alicar rations (Table 1). Nevertheless differences existed with the type of diets as indicated by the slopes of the relationships (Figure 3a). Generally, the ME concentration of low-energy diets (<12.3 MJ/kg DM) was significantly downgraded, whereas that of high-energy diets (>12.3 MJ/kg DM) significantly upgraded. The same applies to the metabolizability values (*q* = ME/GE), which remained unchanged on average, but which were reduced when *q* < 0.67 and increased when *q* > 0.67.

Altogether, for Alicar rations, INRA (2018) modified the linear relationship between ME and DE (intercept and slope) as compared with INRA (2007).

INRA (2018): ME (MJ) = −2.09 + 1.05 DE (MJ) (S = 0.327, *r*^2^_adj_ = 88.99%)
INRA (2007): ME (MJ) = −0.88 + 0.89 DE (MJ) (S = 0.134, *r*^2^_adj_ = 97.48%)

The linear relationships depart from the constant ME/DE value of 0.82 used by NASEM (2016), while the slope of the relationship derived by Galyean et al. (2016) from calorimetry studies was intermediate between INRA (2007) and INRA (2018). These relationships, however, are not interchangeable because of their use in other calculation steps of energy requirements (Galyean et al., 2016; Tedeschi and Fox, 2018).

As a consequence for taking additional dietary factors in the prediction of ME and as also noted by Galyean et al. (2016) and Tedeschi and Fox (2018), the revision of the dietary ME values somewhat modified the dietary ME concentrations in the three selected INRA experiments previously cited. When NE intakes were subsequently calculated from ME, applying the same equations for the efficiency of ME utilization into NE as in INRA (2007) values, but using the revised *q*, values were slightly modified but this was not sufficient to explain the different carcass composition of fattening cattle fed similar NE intakes from contrasted diets (Figure 1b). This suggested that revisiting the accuracy of ME intake was not sufficient and that the influence of the nutrient composition of the ME on the conversion of ME into protein and fat depots also had to be revised as also noted by other systems (e.g., NASEM, 2016).

## PARTITIONING ME INTO NUTRIENTS

To revisit the conversion of ME into NE and investigate the possible influence of nutrient balance on energy depots, ME needs to be decomposed into its constitutive nutrients and nutrient fluxes within the animal have to be evaluated. This section summarizes recent progress made to predict nutrient fluxes within the animal.

### Digestive Fluxes Can Be Used to Decompose the Aggregated ME Units into Metabolizable Nutrients

Recent advances have been made to predict metabolizable, or absorbable, nutrients from individual feed constituents considering their digestibility and the partition of digestion between the rumen and the small and large intestines and to include these predictions in the feeding system. The concept of nutrient fluxes was applied to digestive flows when feed ingredients were decomposed into their constituents with sufficient detail to be quantitatively related to the digestion end products. The nutrients considered are the VFA, glucose, long chain fatty acids, MPs, and metabolizable amino acids. According to INRA (2018) (Figure 2), the production of rumen VFA is linearly related to the quantity of fermented OM (FOM), with an average value of 8.35 moles VFA/kg FOM, with a negative impact of the proportion of concentrate in the diet. Considering the inaccuracy of the classic “per substrate” approach based on stoichiometric coefficients to estimate partition of VFA for each fermented substrate (e.g., in CNCPS, Pitt et al., 1996), an alternative “per VFA” approach based on empirical equations with dietary and digestive factors as explanatory variables has been developed by Nozière et al. (2011) for each VFA and retained in INRA (2018). The molar proportion of individual VFA is derived from the composition of digestible OM (its concentration in digestible NDF), the ruminal starch digestibility, and the feeding level. VFA are also produced in the large intestine; they are assumed to be dependent on OM fermented in the hindgut, with a less variable profile than in the rumen. Amounts of glucose correspond to starch digested in the small intestine and depend on the flow of starch to the duodenum and its digestibility therein. For long chain fatty acids, predictions of absorbed digestible fatty acids depend on the dietary fatty acid concentration and their intestinal digestibility.

### Predicted Metabolizable Nutrients were Evaluated Against Net Portal Nutrient Fluxes

**Figure 4.**
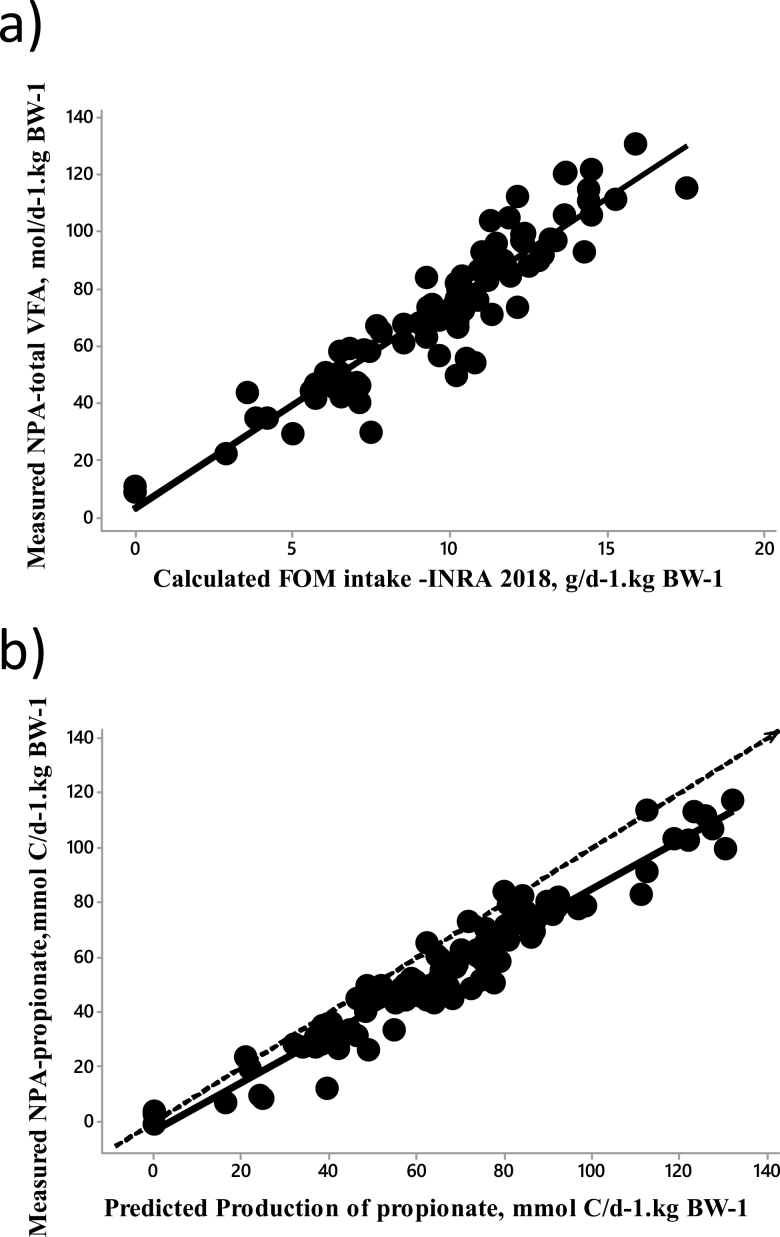
Metabolizable nutrients. (a) Relationship between measured NPA of total VFA and the FOM intake calculated according to INRA (2018). (b) Relationship between measured NPA of propionate and production of propionate in the rumen and the hindgut predicted according to INRA (2018). The Flora database was used.

Metabolizable nutrients, predicted as described above, were evaluated against measured net portal appearance (NPA) of nutrients using the Flora database (Vernet and Ortigues-Marty, 2006) as described by Nozière et al. (2016; Figure 4). All ingredients of experimental diets were characterized according to INRA (2018) for subsequent calculation of metabolizable nutrients, using the systool.fr application (Chapoutot et al., 2015). This concerned flows of acetate, propionate, butyrate, and minor VFAs produced in rumen and the hindgut, absorbed glucose (i.e., from starch apparently digested in the small intestine), and total amino acids truly absorbed in the small intestine (i.e., MP, expressed in protein digestible in the intestine [PDI]). Those calculated fluxes of metabolizable nutrients were compared with their measured NPA, and the biological consistency of the relationship was assessed. As expected, calculated absorbable flows were highly correlated to their measured NPA, and the relationships were consistent with current quantitative knowledge on metabolism of portal-drained viscera. For glucose, the intercept was negative, reflecting net basal uptake of glucose by portal-drained viscera with nonstarchy diets, and the slope averaged 0.40, which is consistent with net portal recovery of glucose provided by duodenal perfusions (Kreikemeier et al., 1991). For VFAs and amino acids, the intercept did not differ from 0. The portal recovery for acetate (corrected for uptake of arterial supply by portal-drained viscera; Nozière and Hoch, 2005) and propionate, which are known to be poorly metabolized by portal-drained viscera (Kristensen and Harmon, 2006), was higher than 75%, whereas NPA of butyrate and β-hydroxybutyrate averaged 32% and 54% of calculated absorbable butyrate flow. Measured NPA of N from amino acids (assumed to be 1.37 × α-amino-N; Martineau et al., 2014) averaged 68.5% of N from calculated MP, which is close to the average efficiency of use of MPs (Sauvant et al., 2015). Interestingly, for all nutrients, relationships were not affected by a study effect, and among dietary or animal characteristics, only few were correlated to residuals and none significantly improved the models.

### Predicted Metabolizable Nutrients are Quantitatively Compatible with ME

**Figure 5.**
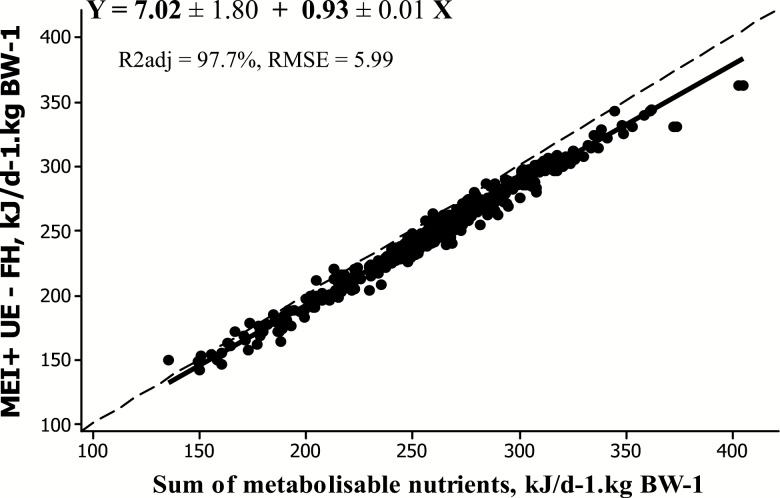
Comparison between two expressions of absorbed energy, calculated from INRA (2018) using the Flora database.

To subsequently evaluate if the INRA (2018) predictions of metabolizable nutrients are quantitatively compatible with predictions of ME, we used the concept of absorbed energy (AE) used by Gill et al. (1984) and Baldwin et al. (1987) and refined by Loncke et al. (2011). AE can be defined in two ways (Figure 2). First, from ME, with AE = DE − the energy lost as methane (CH4-E) − heat of fermentations (HF). Since ME = DE − CH4-E − urinary energy (UE), the former expression can be written as AE = ME + UE − HF. Second, on physiological bases as the sum of all metabolizable nutrients (expressed on an energy basis). As a result, ME can be decomposed in fluxes of nutrients if the sum of all metabolizable nutrients equals “ME + UE − HF.” This hypothesis was tested using the Alicar database. Flows of metabolizable nutrients and CH4-E were calculated as in INRA (2018). In absence of data on HF and on its variation across a whole range of diets, HF was assumed to be equal to UE losses as in Loncke et al. (2011). On average, AE calculated as the sum of metabolizable nutrients was only 4.5% lower than AE calculated as “metabolizable energy intake + UE – HF.” There was a good linear fit between the two variables with a slope (0.93) still significantly different but closer to 1 as compared with INRA (2007) and an intercept significantly different from 0. The departure from the bissectrice is attributed to errors of prediction. The relationship (Figure 5) was improved as compared with that derived using INRA (2007) and presented by Loncke et al. (2011), probably as a result of digestive interactions being now accounted for in INRA (2018). This step shows that ME can be decomposed into metabolizable nutrients with a higher accuracy than using INRA (2007).

This step forward is critical as it conditioned the possibility of quantitatively combining fluxes of energy between the animal and its environment with fluxes of nutrients between tissues and organs within the animal.

### Prediction of Nutrient Fluxes between Tissues and Organs

Progress on the empirical prediction of nutrient fluxes between tissues and organs has also been achieved (Figure 2). Net absorption of nutrients in the portal vein (acetate, propionate, butyrate, minor VFAs, α-amino-N, ammonia, glucose, L-lactate, and β-hydroxybutyrate) is predicted from a combination of dietary characteristics that meet the same criteria of pragmatism and empirism as imposed in the revision of the INRA feeding system (Loncke, 2009; Loncke et al., 2009b, 2015). Net nutrient fluxes across the liver (acetate, propionate, butyrate, α-amino-N, ammonia, urea, glucose, L-lactate, and β-hydroxybutyrate) are themselves predicted from net portal fluxes, and the net release of nutrients from the visceral tissues (portal-drained viscera and liver) can thereby be predicted from dietary characteristics (Loncke, 2009; Loncke et al., 2015). Energy expenditure of the portal-drained viscera and the liver are also predicted from dietary characteristics (Loncke et al., 2009a; Al-Jammas et al., 2013). Equations are based on the principle of mass action laws. Predictions were developed for net fluxes, that is, incremental fluxes rather than the absolute afferent and efferent fluxes. The rationale was to quantify the major driving forces of uptake or release of nutrients from the portal-drained viscera and the liver, when a daily ration is supplied to an animal in a steady state. The empirical models aim at partitioning the major energy fluxes arising from a daily ration between tissues and organs, irrespective of the amount of nutrients already circulating in the animal from previous rations.

Some secondary driving forces are also accounted for through the factors of variation of the relationships. By contrast, driving forces that reflect the dynamic changes, the initial nutritional status of the animal, and the influence (Hanigan, 2005) of the arterial or venous origin of nutrients and its impact on nutrient fate are not accounted for. The approach is static; it is a simplification of nutrient fate but compatible with current feeding systems that are also static. Predictions apply to all ruminant species, and the time unit is the day.

Figure 2 illustrates this approach. For example and as summarized by Ortigues-Marty et al. (2010), the amounts of the individual VFA (acetate, propionate, and butyrate) absorbed at portal level are derived from the amount of FOM and its composition in rumen digestible NDF. Uptake of those nutrients by the liver is calculated from their portal appearance according to linear responses. The resulting net splanchnic release of the secondary metabolites (β-hydroxybutyrate, glucose, and L-lactate) is linearly or quadratically derived from the amount of FOM and its composition in rumen digestible NDF, the amounts of starch digested in the rumen or in the intestine, of MPs, and the energy balance of the animals.

Limits in the accuracy of these prediction equations are recognized. They originate from the uncertainty associated with the experimental measurements (Rodríguez-López et al., 2014, 2016) from the limited, yet exhaustive, data set used for their development and the use of both cattle and sheep data.

The set of prediction equations constitutes a milestone to combine the aggregated energy fluxes with their subcomponent nutrient fluxes at tissue level. Ultimately, for fattening cattle, they should be completed with the evaluation of nutrient fluxes in and out of the carcass or of the tissues of economic interest (muscle and adipose tissues). Unfortunately available data are currently too limited.

## REVISITING THE CONVERSION OF ME INTO NE

Previous developments opened the possibility of revisiting the NE value of diets, in particular, the prediction of ME deposited as NE during growth and fattening and testing its validity.

### Overview of the Conversion of ME into NE in Different Feeding Systems

It is generally assumed that animal type (genetics and sex), physiological age, and rate of growth determine the amounts of fat and protein and, hence, energy deposited and that dietary components determine the efficiency of ME use for energy deposition (Tedeschi et al., 2010). Still, in different feeding systems, models were developed to evaluate the NE value of diets of growing and fattening cattle, the NE requirements of animals, and the linkage between both.

In the INRA (1978, 2007, 2018) NE system, the NE value of diets is calculated from dietary ME concentrations and efficiency coefficients “*k*,” which are defined as NE/ME ratios and formulated as linear functions of the metabolizability of the diet “*q* = ME/GE” and the feeding level as summarized in INRA (1978) and kept unchanged in INRA (2018). Efficiencies of ME utilization into NE are derived from measurements made by indirect calorimetry. The majority of measurements used to quantify *k* values were done in adult fattening animals. As a result, the NE intake evaluated from the NE concentrations of diets does not necessarily match the energy gain of the animals measured at slaughter because the composition of the energy gain varies with animal type and BW and because the influence of diet composition on tissue gain varies with the physiological age of the animal. By contrast, NASEM (2016) determines the NE value of diets from comparative slaughter measurements done on a wide range of animals, fed different diets at different levels of intake (NASEM, 2016). These differences result in divergent estimations of dietary NE values (Ferrell and Oltjen, 2008; NASEM, 2016).

On the other hand, NE requirements of animals are evaluated from modeled fat and protein gain, assuming that the chemical or tissue composition of the whole body only depends on animal type, BW, and physiological age (NASEM, 2016), as well as ADG (INRA, 2007, 2018). Because INRA (2007) NE requirements for gain were defined using a different methodology than that used for the NE value of diets, a conversion step was adopted as summarized in INRA (2018) and was kept unchanged in the recent update of the system.

Whatever the approach, the current energy systems for fattening cattle use coefficients of conversion of energy to productive processes, which do not simultaneously account for all sources of variability (Tedeschi et al., 2010; NASEM, 2016). Developing relationships among the productive processes and the quantity of nutrients required to support those functions would be a step forward.

### Investigating the Relationship between ME and Carcass Composition

To test whether the INRA (2007) calculation of the NE values of diets from ME reflects the relationship between ME and carcass composition, availability of data has been considered. In beef cattle, experiments that have quantified NE gains remain scarce. Measurements of daily gains and composition of whole BW gain are costly and time demanding and require determination of body composition in a representative initial slaughter group at the onset of the experiment and in other groups at the end. Instead, we considered the composition of the carcass. Furthermore, because in INRA (2007, 2018) the conversion of ME into NE depends on the efficiency *k* values and, hence, on the metabolizability of the diet “*q*” and the level of intake, we investigated the linearity of the relationship between the metabolizability of the diet (expressing diet composition) and the composition of carcass at similar intake levels. We tested if the metabolizability of the diet “*q*” correctly reflects the impact of diet composition on carcass composition for a given type of fattening cattle and a close range of BWs at similar ME intake.

**Figure 6.**
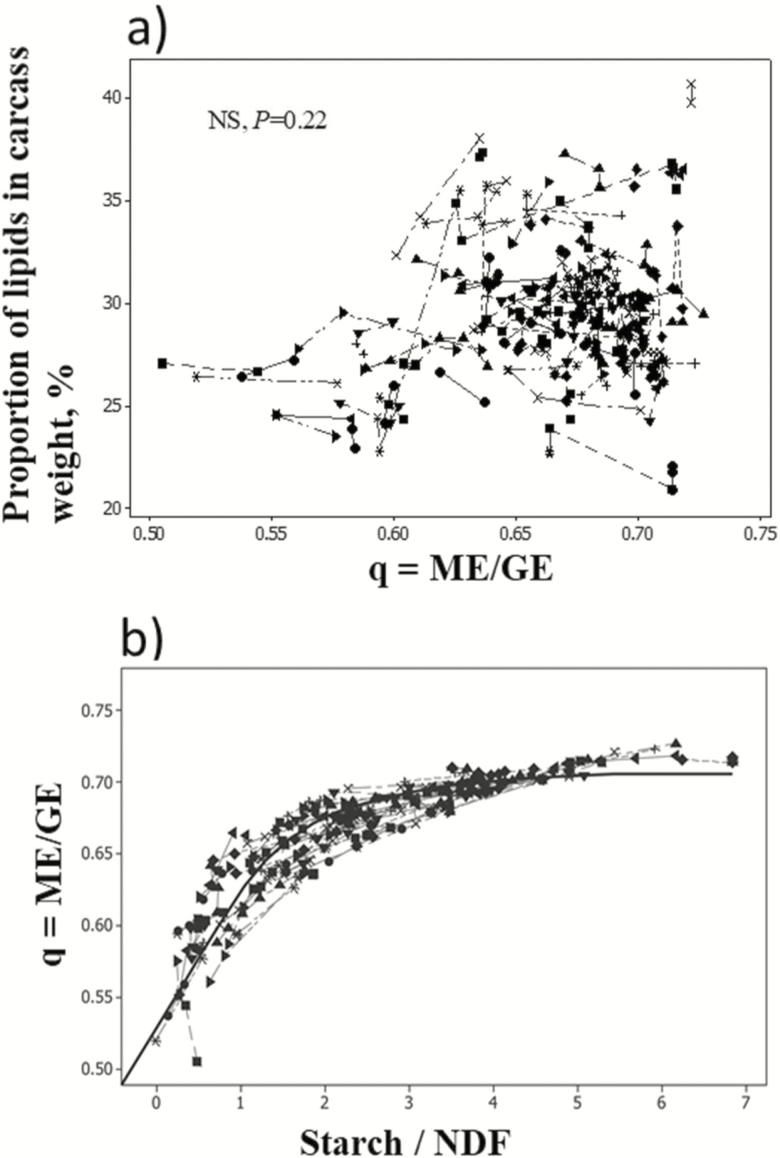
Relationships between (a) the proportion of lipids in the carcass of fattening cattle and the metabolizability of diets (b) and the metabolizability of diets and the dietary starch to NDF concentration.

**Figure 7.**
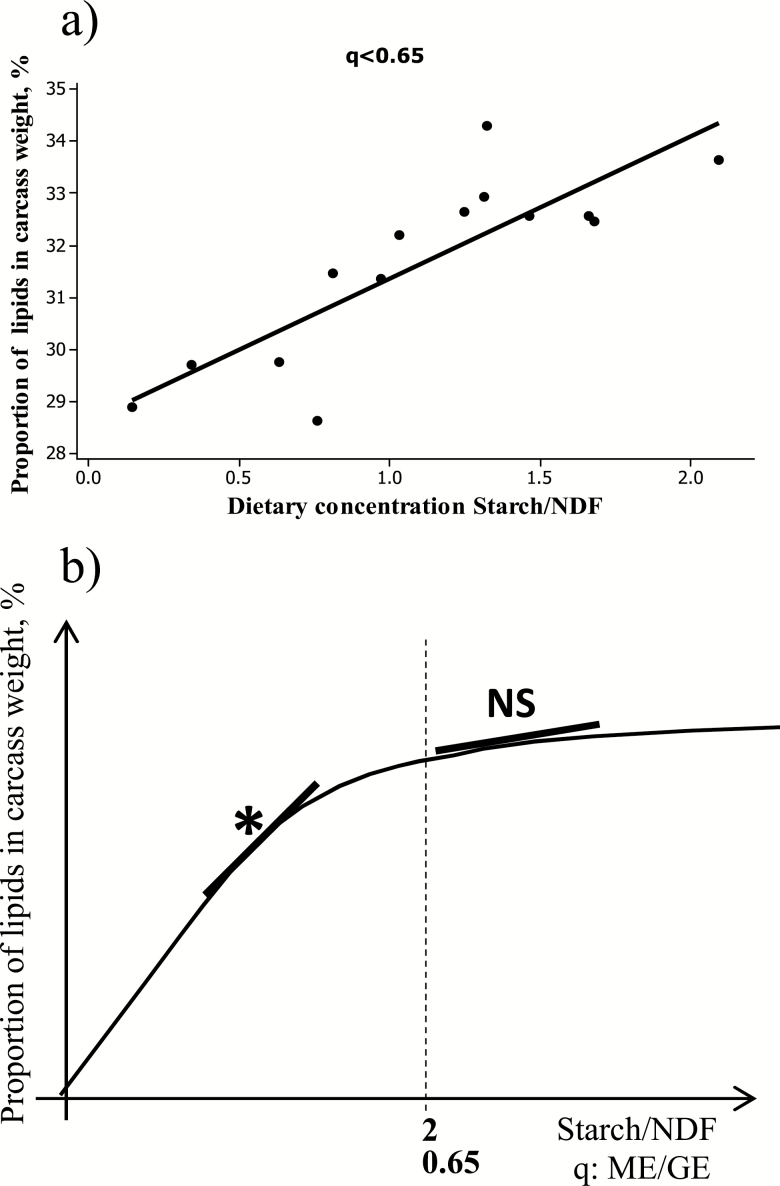
(a) Relationships between the proportion of lipids in the carcass of fattening cattle and the dietary starch to NDF concentration ratio for diets with a metabolizability inferior to 0.65. Relationship was not significant when metabolizability was superior to 0.65. Data were from the Alicar database. (b) Schematic representation of carcass lipid changes in fattening cattle with diet composition.

The Alicar database was used. Studies with similar ME intake across treatments were selected and an intrastudy meta-analysis was conducted to account for breed and animal differences (Al-Jammas, 2017). No significant relationship was found between *q* and the proportion of lipids in the carcass (Figure 6a). Instead a wide dispersion of data was noted and further explored. Metabolizability is supposed to reflect the energy density of the diet, which itself varies, in particular, with the starch/NDF concentration ratio of the diet. A curvilinear relationship was found between *q* and the dietary starch/NDF ratio (Figure 6b), raising the hypothesis that *q* is nonlinearly related to carcass composition. Evidence was obtained after separating data by hierarchical classification (Arabie et al., 1996). For diets with *q* < 0.65 or a dietary starch/NDF ratio <2, the proportion of lipids in the carcass was significantly and positively linearly related to the starch/NDF ratio of diets (Figure 7a). No significant relationship was obtained for diets with *q* > 0.65 or a dietary starch/NDF ratio >2. Unfortunately, the latter group mostly included results from experiments conducted in the United States with animals fed digestive or metabolic modifiers, which may modify the dietary ME concentration of diets and/or metabolic fate of nutrients, thereby influencing fat depots (e.g., Byers, 1980; NASEM, 2016) and potentially biasing the conclusions. Confirmation is needed but will depend on new data becoming available for animals of Continental breeds fed high-energy-dense diets without any digestive or metabolic modifiers.

These results suggest that the proportion of lipids in the carcass varies nonlinearly with the starch/NDF ratio or the *q* value of the diet as schematically represented Figure 7b, showing an increase in diets with *q* < 0.65 or starch/NDF <2, followed by a plateau indicative of a maximum in the proportion of lipids deposited in the carcass. As indicated in Figure 1b, at similar NE intake, the highest proportions of lipids in the carcass were measured for animals fed corn or grass silages and the lowest proportions for animals fed hay or haylage. These exploratory results are insufficient to develop relationships between ME intake, dietary factors, and carcass composition. They suggest, however, that the balance of nutrients derived from a ration of medium to low energy density might influence the composition (lipid and protein) of the energy deposited in the carcass. The glucogenic to cetogenic balance of nutrients absorbed in the portal vein was shown by principal component analysis to significantly explain the proportion of lipids in the carcass only when diets had a metabolizability value below 0.65. If confirmed, not only at carcass but also at whole body level, these results could have major implications as *q* values of fattening diets in France average 0.62, positioning those diets in dietary conditions affecting the proportion of fat depots. Implications would be most important for fattening practices favoring forage-based diets instead of diets based on human-edible feeds. They would also imply that the *k* values might not be directly related to potential differences in the composition of gain. This work was conducted with the INRA (2007) metabolizability values. If INRA (2018) metabolizability values that account for the impact of digestive interactions due to intake and forage to concentrate ratio were used, the same conclusions would apply because of the linear relationship between INRA (2018) and INRA (2007) dietary ME concentrations.

### Nutrient Fluxes Can Contribute to Revise the Relationship between ME and Carcass Composition

A growth model (MecSic; Hoch and Agabriel, 2004) was used to determine if nutrient fluxes could improve the predicted response of carcass composition to ME intake when the composition of ME in nutrients varies. MecSic simulates the growth of protein and lipid masses in the carcass as well as in the noncarcass according to Gompertz equations and uses daily ME intake as the single driving variable. According to Oltjen et al. (1986), ME availability, physiological age, and adult protein mass drive protein deposition, independently of dietary and ME composition. As a result, MecSic was built on the hypothesis that the dynamics of growth, whether restricted or in compensatory periods, is the most important driver of carcass composition during the rearing period. Parameters are adjusted for the type of animals. MecSic, however, does not correctly simulate adipose tissue growth of finishing animals fed isoenergetic supply from different diets (forage vs. concentrate based) and growing continuously (Garcia et al., 2008). Attempts were made to upgrade MecSic using criteria indicative of nutrient balance. From a limited number of published studies, Agabriel et al. (2013) identified a nutrient balance criteria accounting for the acetate, lipid, and amino acid fluxes at portal level that could be used to modulate the Gompertz function for lipid carcass synthesis. Al-Jammas (2017) tested whether the glucogenic to cetogenic balance of nutrients (or its proxy, the dietary starch to NDF concentration ratio) could explain the variability of MecSic parameters adjusted for the different finishing diets of the INRA experiments presented in Figure 1, all of which had metabolizability values below 0.65. Results indicated that after adjustment, the parameter that expresses the synthesis rate of lipids in the carcass varied with the proxy. These proxies now need to be evaluated using more data, and the concept can be further developed searching for additional criteria of nutrient balance and introducing them in MecSic to account for the influence of diet composition on lipid deposition in the carcass and whole body at similar ME intakes. Results show that consideration of nutrient fluxes can be achieved without introducing unnecessary complexity in the NE feeding systems. Simple empirical proxies of nutrient fluxes can be developed limiting the use of complex mechanist modeling.

### Further Perspectives

Nutrient fluxes at tissue level offer other perspectives. For example, as proposed by INRA (2018), composition of fatty acids in muscles, including polyunsaturated fatty acids, may vary with diets and the proportion of C18:3-n-3 in diets. Accounting for the fluxes of individual metabolizable fatty acids might improve the prediction of the nutritional value of meat.

Also at tissue level, predicted nutrient fluxes in and out of a tissue can be used to adjust a model of nutrient use within the tissue at steady state. Basically, the quantitative aspects of nutrient flux in and out of a tissue (level i) can set limits for the nutrient transactions within the tissue (level i-1) and be used to fit parameter values (Thornley and France, 2007). The principle is being applied for liver metabolism (Bahloul et al., 2012) and could be applied to other tissue compartments.

A more physiological approach to energy fluxes may enable reevaluation of the nonproductive expenditures of energy required to maintain the structure and integrity of the animal body (Blaxter, 1962). In practice, maintenance energy requirements are evaluated in standardized conditions and applying standardized statistical calculations, the diversity of which has evolved over time to fit different purposes (Blaxter, 1989; Cannas et al., 2010) and resulted in a diversity of values (INRA, 2018). Maintenance, however, does not necessarily reflect the energy required for the nonproductive functions such as that required by the internal organ. Indeed, on average energy expenditure by visceral organs altogether represents 0.4-fold the maintenance ME requirements for nonproductive adults and 0.6-, 0.5-, and 1.2-fold for growing, gestating, and lactating ruminants, respectively (Ortigues-Marty et al., 2017). A more physiological approach to the nonproductive energy requirements could account for the energy expenditure of visceral organs derived from oxygen fluxes at tissue level, which can be predicted along with the nutrient fluxes.

## CONCLUSIONS

Partitioning energy fluxes into their subcomponent (nutrient) fluxes, more physiologically based, is possible and compatible with the traditional approach. Benefits are to combine on a quantitative basis the traditional production-driven approach, which has proved its robustness and which will remain, with a physiologically driven one, which can supply indicators of prediction of some animal responses. Pools of energy considered include the relatively easy to measure ones at whole animal level (feces, urine, CH4, O2, and CO2) and the predicted flows at digestive and tissue levels that are more difficult to measure (digesta flows, nutrients fluxes between and ultimately within tissues). The added value of considering nutrient fluxes is several fold: a better accuracy in the evaluation of the DE and ME concentrations of diets; for fattening cattle, the perspective to refine NE recommendations accounting for forage rich diets; and the perspective to develop nutrient-based response equations of performance and carcass composition that integrate nutrient interactions at digestive and metabolic levels. Combining the empirical nutrient fluxes at tissue level with energy fluxes at whole animal level has potential to refine the NE system for growing and fattening cattle, such as in the prediction of carcass composition, of fatty acid composition of meat, and more generally to adjust tissue metabolism models or contribute to the definition of the nonproductive energy requirements.
